# NUP153 immunoreactivity in neuroendocrine neoplasms supports the diagnosis of NET versus NEC and is modulated by post‐translational modifications

**DOI:** 10.1111/jne.70240

**Published:** 2026-08-12

**Authors:** Sven Mattern, Arslan Ali, Vanessa Hollfoth, Eyyub Bag, Katharina Kluthe, Esther Herpel, Frank Bergmann, Thomas Muley, Michael Meister, Judith Lehmann‐Koch, Benjamin Goeppert, Arne Warth, Kai Breuhahn, Irina Bonzheim, Stephan Singer, Kerstin Singer

**Affiliations:** ^1^ Institute of Pathology University Hospital Tübingen Tübingen Germany; ^2^ Clinical Pathology Darmstadt Hospital Darmstadt Germany; ^3^ Institute of Pathology University Hospital Heidelberg Heidelberg Germany; ^4^ Translational Research Unit Thoraxklinik at University Hospital Heidelberg Heidelberg Germany; ^5^ Translational Lung Research Center (TLRC) German Center for Lung Research (DZL) Heidelberg Germany; ^6^ Institute for Pathology SLK‐Kliniken Heilbronn Heilbronn Germany; ^7^ Institute for Pathology and Neuropathology RKH Hospital Ludwigsburg Ludwigsburg Germany; ^8^ ÜGP MVZ Institute for Pathology, Dermatopathology, Cytology, and Molecular Pathology GbR Wetzlar Germany; ^9^ ENETS Center of Excellence University Hospital Tübingen Tübingen Germany

**Keywords:** neuroendocrine neoplasms (NEN), nuclear pore complex (NPC), NUP153, post‐translational modifications (PTM)

## Abstract

Nucleoporins (NUPs) constitute the nuclear pore complex (NPC) and are essentially involved in nuclear transport, chromatin organization, and context‐dependent gene regulation. However, the role of NUPs in neuroendocrine (tumor)biology and related diagnostic potential is poorly defined. In this study, we comparatively analyzed immunohistochemical expression patterns of NUP98 and NUP153 (sharing structural and functional similarities) across a large variety of human tissues and tumors (total *N* > 600), with a focus on neuroendocrine neoplasms (NENs (*n* = 361)). While both NUPs showed a nuclear rim accentuated staining pattern, NUP98 exhibited ubiquitous and NUP153 a striking cell‐ and tissue/tumor‐type‐dependent immunoreactivity. More specifically, NUP153 immunoreactivity was consistently detectable in neuroendocrine tissues (e.g., pancreatic islets) and retained in neuroendocrine tumors (NETs) of the pancreas, lung, appendix, and small intestine, but almost completely absent in neuroendocrine carcinomas (NECs) and non‐neuroendocrine carcinomas. Loss of NUP153 staining correlated with poorer clinical outcomes in pancreatic NETs. Further analyses revealed that NUP153 messenger ribonucleic acid (mRNA) and total protein levels were not significantly different between NETs and non‐neuroendocrine carcinomas, as evaluated by quantitative real‐time polymerase chain reaction and targeted proteomics. Differential isoform usage was ruled out by polymerase chain reaction and sequencing as another possible explanation for the discriminative immunohistochemical findings. Finally, a high density of post‐translational modifications (PTMs) within the antigen sequence could be discovered and indicated PTM‐dependent detection as the likely cause of differential staining. Collectively, our findings suggest that NUP153 is a differentiation‐dependent and PTM‐sensitive immunohistochemical marker in NENs with diagnostic and prognostic potential. It also emphasizes the importance of orthogonal molecular analyses for correct interpretation of IHC stainings.

## 1 INTRODUCTION

The nuclear pore complex (NPC) is embedded in the nuclear envelope and consists of ~30 different nucleoporins (NUPs) forming different subcomplexes.^1^, ^2^, ^3^, ^4^, ^5^ The NPC is the only trafficking gateway between the cytoplasm and the nucleus and is thus a pivotal component of the nucleo‐cytoplasmic transport machinery. However, there are also transport‐independent functions of NUPs, such as chromatin interactions and gene regulation indicating several levels at which cancer‐relevant processes can be linked to the NPC (e.g., References [^6^, ^7^, ^8^]). Several NUPs have been documented to play a role in cancer, including nucleoporin‐98 (NUP98) and nucleoporin‐153 (NUP153),^8^, ^9^ which also share functional and structural similarities. Both NUPs exhibit phenylalanine‐glycine repeat (FG‐repeat) domains and NPC mobility in a transcription‐dependent manner.^10^, ^11^, ^12^

NUP98 appears to play diverse roles in tumor biology. In leukemias, NUP98‐containing fusion proteins are oncogenic and occur as the result of chromosomal translocations (e.g., References [^13^, ^14^]) with recent work showing that NUP98‐lysine‐specific demethylase 5A (KDM5A) fusions form aberrant condensates and create a targetable Cyclin Dependent Kinase 12 (CDK12)‐dependency.^15^ On the other hand, NUP98 has been shown to be involved in P53 target gene selection in hepatocellular carcinoma (HCC)^7^ and also forms off‐pore condensates that mobilize heterochromatic breaks to prevent error‐prone repair, linking its transport and genome‐stability associated functions to tumor biology.^16^

For NUP153, upregulation and association with tumor progression were shown in gastric cancer.^9^ In HCC NUP153 drives proliferation via myc proto‐oncogene protein (MYC)^17^ and in urothelial carcinoma as well as retinoblastoma NUP153 elevation correlates with migration/proliferation.^18^ In squamous epithelium NUP153 tethers super‐enhancers at the pore to sustain transformation‐related protein 63 (*TP63*) expression, which in turn affects differentiation.^19^ Moreover, NUP153 is involved in deoxyribonucleic acid (DNA) repair by mediating nuclear import of 53BP1.^20^ Together, these findings underscore the diverse and context‐dependent tumor‐modulatory functions of NUP98 and NUP153. However, their roles in neuroendocrine differentiation and neoplasms remain virtually unexplored.

Neuroendocrine neoplasms (NEN) represent a heterogeneous group of tumors^21^ with increasing incidence, diverse tumor biology, prognosis, and therapeutic options.^22^, ^23^ NEN derive from cells of the diffuse or organ‐specific neuroendocrine system (mainly in the gastroenteropancreatic (GEP) organ system and the lung) and are often diagnosed in advanced/metastasized stages. The diagnosis of NEN requires histological evaluation with the prognostically and therapeutically relevant categorization into either well‐differentiated neuroendocrine tumors (NET, G1–G3) or poorly differentiated neuroendocrine carcinomas (NEC), depending on morphological criteria, the proliferation marker protein Ki‐67 (MIB1) index, and/or mitotic rate.^24^, ^25^

Here, we comparatively screened a large variety of human tissues and tumors for NUP98 and NUP153 by immunohistochemistry (IHC). NUP98 was ubiquitously positive in all tissues tested, whereas NUP153 immunoreactivity was restricted to a small subset of cell types, tissues, and tumors. Notably, neuroendocrine cells and NETs showed strong NUP153 staining, which correlated with overall survival, while NECs and non‐NECs were nearly completely negative. Targeted proteomic, quantitative real‐time polymerase chain reaction (qPCR), and sequencing analyses indicated that this difference is unrelated to total NUP153 protein levels or isoform expression, suggesting instead a cell‐ and tumor‐type‐specific post‐translational modification affecting NUP153 immunoreactivity.

## 2 MATERIALS AND METHODS

### 2.1 Patient samples and tumor specimens

All tissue specimens were provided by the tissue bank of the National Center for Tumor Diseases (Heidelberg, Germany; ethics proposal 206/2005, University of Heidelberg), unless otherwise specified. Informed consent was obtained from all patients and/or their legal guardian(s). All methods were carried out in accordance with relevant guidelines and regulations. For further information, see Supporting Information S1.

### 2.2 Patient samples used for sequencing analyses and full‐section slides

All tissue specimens were provided by the biobank of the Comprehensive Cancer Center Tuebingen—Stuttgart, Germany (Tuebingen, Germany; ethics proposal 508/2016B01, University of Tuebingen). Informed consent was obtained from all patients and/or their legal guardian(s). The study protocol was approved by the local Ethics Committee of the University of Tuebingen (Project Number 032/2021B02). All methods were carried out in accordance with relevant guidelines and regulations.

### 2.3 Patient samples used for immunoblotting, mass spectrometry, and qPCR

The tissue specimens were provided by the tissue bank of the National Center for Tumor Diseases (Heidelberg, Germany; ethics proposal 206/2005, University of Heidelberg). For further information, see Supporting Information S1.

### 2.4 Immunohistochemistry and tissue microarray analysis

The immunohistochemical staining of nuclear NUP153 and NUP98 was semi‐quantitatively evaluated. For further information, see Supporting Information S1.

### 2.5 Gel electrophoresis and immunoblotting

Protein extracts were isolated from frozen tissue samples using the mixer‐mill MM 200 with steel grinding jars and 5 mm steel grinding balls (Retsch). For further information, see Supporting Information S1.

### 2.6 Total RNA isolation, cDNA synthesis and quantitative real‐time polymerase chain reaction

For sample preparations of total RNA from lung carcinoids and NSCLCs, 10–15 cryosections (10 μm each) were homogenized with the TissueLyser mixer‐mill disruptor (2 × 30 s, 3.00 Hz; Qiagen). For further information, see Supporting Information S1.

### 2.7 NUP153 messenger ribonucleic acid isoform analysis

RNA was extracted from macrodissected 5 μm paraffin sections using the Maxwell® RSC RNA FFPE Kit and the Maxwell® RSC Instrument (Promega, Madison, WI, USA) according to the manufacturer's instructions. For further information, see Supporting Information S1.

### 2.8 Analysis by targeted proteomics

The tumor tissues from lung carcinoids and NSCLCs were homogenized in urea buffer (10 M urea in 250 mM NH_4_HCO_3_) and 2% (w/v) Rapigest (Waters, Eschborn, Germany) reaching a final concentration of 4 M and 0.2%, respectively (w/v), aided by three sonication steps of 30 s each in a VialTweeter, interrupted by cooling down on ice for 1 min between each step. Reduction of solubilized proteins was achieved by using 10 mM dithiothreitol (DTT) (at 37°C for 30 min). The addition of 15 mM iodoacetamide for 30 min in the dark resulted in the alkylation of cysteine residues. For the enzymatic digestion, samples were incubated with LysC (Wako Chemicals, Neuss, Germany) in an enzyme to protein ratio of 1:100 (w/w) for 4 h at 37°C. Followed by the addition of trypsin (Promega, Mannheim, Germany) in a ratio of 1:50 (w/w) for overnight digestion at 37°C. After digestion, samples were acidified with 10% (v/v) trifluoroacetic acid (TFA) to allow for the cleavage of Rapigest at 37°C for 30 min. After centrifuging the samples (17,000 × *g* for 5 min at room temperature (RT)), samples were desalted using C18 spin columns (Harvard Apparatus, Holliston, MA, USA) according to the recommended procedure by the manufacturer. For further information, see Supporting Information S1.

### 2.9 Statistical analysis and software

Analyses were performed using Excel LTSC MSO Version 2408 (Microsoft, Redmond, WA, USA; https://www.microsoft.com/microsoft-365/excel) and Graphpad Prism 10.1.1 (https://www.graphpad.com/prism/). For further information, see Supporting Information S1.

## 3 RESULTS

### 3.1 NUP98 exhibits ubiquitous, while NUP153 shows cell‐type‐specific immunoreactivity in human tissues

**FIGURE 1:**
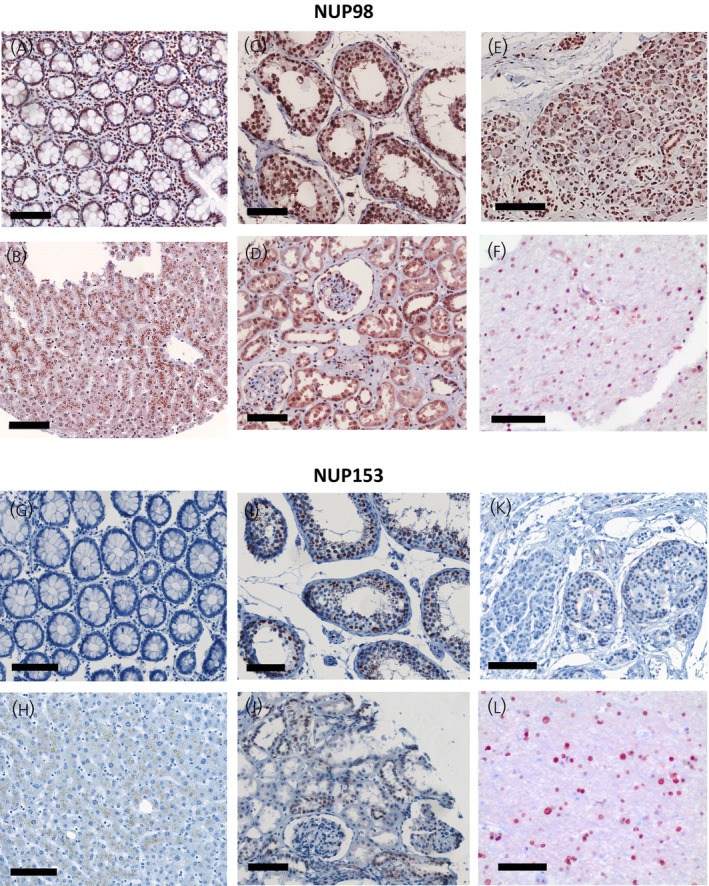
Comparative immunohistochemical stainings of nucleoporin‐153 (NUP153) and nucleoporin‐98 (NUP98) in a variety of normal tissues. Immunohistochemistry (IHC) of NUP98 in representative tissue specimen of (A) colon, (B) liver, (C) testis, (D) kidney, (E) pancreas, (F) brain. IHC of NUP153 in representative tissue specimen of (G) colon, (H) liver, (I) testis, (J) kidney, (K) pancreas, (L) brain. Scale bar corresponds to 100 μm.

To study differences and/or similarities of NUP98 and NUP153 immunoreactivity in various tissues we performed respective immunohistochemical stainings (IHC) of human pan‐normal tissue microarrays (TMAs) containing approximately 200 samples derived from 25 tissues (composition of the PAN‐tissue‐TMA is detailed in Tables S1 and S2, Figure 1).

NUP98 showed a strong ubiquitous immunoreactivity in all tissues being tested with no apparent cell type specificity as exemplified for colon, liver, testis, kidney, pancreas, and brain in Figure 1A–F and Table S1. In contrast, the immunohistochemical NUP153 staining (polyclonal HPA27896) was negative in a large fraction of tissues, including colon and liver (Figure 1G,H). However, a subset of cell types in particular tissues showed positive NUP153 staining such as testis, kidney, pancreas, and brain (Figure 1I–L). More specifically, in the kidney, we detected a strong positive staining only in the cells of the distal part of the nephron segment (Figure 1J) and in the testis in Sertoli cells (Figure 1I). Interestingly, in the pancreas we found the islets of Langerhans, representing the neuroendocrine compartment of the pancreas, to be positive for NUP153 in contrast to the negativity of the exocrine pancreatic tissue (Figure 1K). These data indicate that despite functional and structural similarities NUP98 and NUP153 show a strikingly different immunoreactivity across human tissues with a selective positivity of NUP153 in neuroendocrine cells (among others).

### 3.2 NUP153 immunoreactivity is maintained in intestinal well‐differentiated neuroendocrine tumors compared to corresponding non‐neuroendocrine carcinomas

**FIGURE 2:**
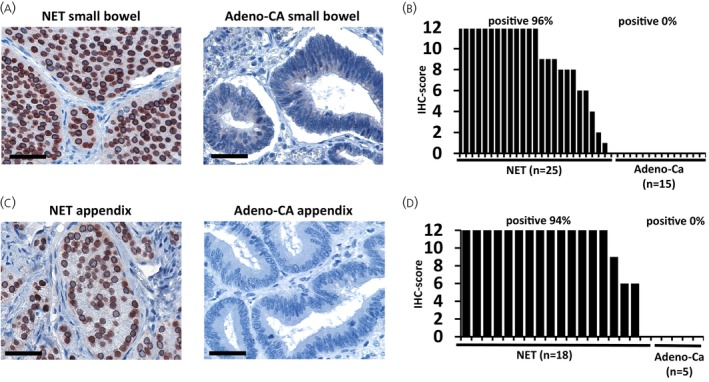
Nucleoporin‐153 (NUP153) immunopositivity is strongly present in small bowel and appendiceal neuroendocrine tumors, but absent in adenocarcinomas of the same organs. (A) Immunohistochemistry (IHC) of NUP153 in representative tissue specimen of neuroendocrine tumors (NET) (left panel), and adenocarcinomas (right panel) of the small bowel. Scale bar, 60 μm. (B) Immunohistochemical scores of NUP153 staining in 25 NETs and 15 adenocarcinomas of the small bowel. (C) IHC of NUP153 in representative tissue specimen of NET (left panel), and adenocarcinomas (right panel) of the appendix. Scale bar, 50 μm. (D) Immunohistochemical scores of NUP153 staining in 18 NETs and five adenocarcinomas of the appendix.

Following up on the observed NUP153 positivity of neuroendocrine cells in normal tissue, we tested if NUP153 immunoreactivity is preserved in neuroendocrine neoplasms (NENs). To do so, we compiled TMAs containing neuroendocrine and non‐neuroendocrine neoplasms of various organs and performed again NUP153 IHC. Overall, we evaluated >400 human tumor samples from organs/tissues in which NENs frequently arise such as the appendix vermiformis, small bowel, pancreas, and lung. In the small bowel 96% of NETs (24/25) were characterized by a strong NUP153 staining, whereas the corresponding small bowel adenocarcinomas (*n* = 15) were completely negative (Figure 2A,B). The nuclear NUP153 staining was accentuated at the nuclear envelope consistent with the predominant localization of an NPC component (Figure 2A). A similar differential IHC staining pattern of NUP153 occurred in NET and adenocarcinomas of the appendix vermiformis with a strong positivity in 94% of the former (17/18) compared to 0% (0/5) of the latter (Figure 2C,D). We conclude that well‐differentiated NETs preserve immunohistochemical NUP153 positivity (HPA27896).

### 3.3 NUP153 immunoreactivity is correlated with tumor cell differentiation and aggressiveness in pancreatic and pulmonary NEN

**FIGURE 3:**
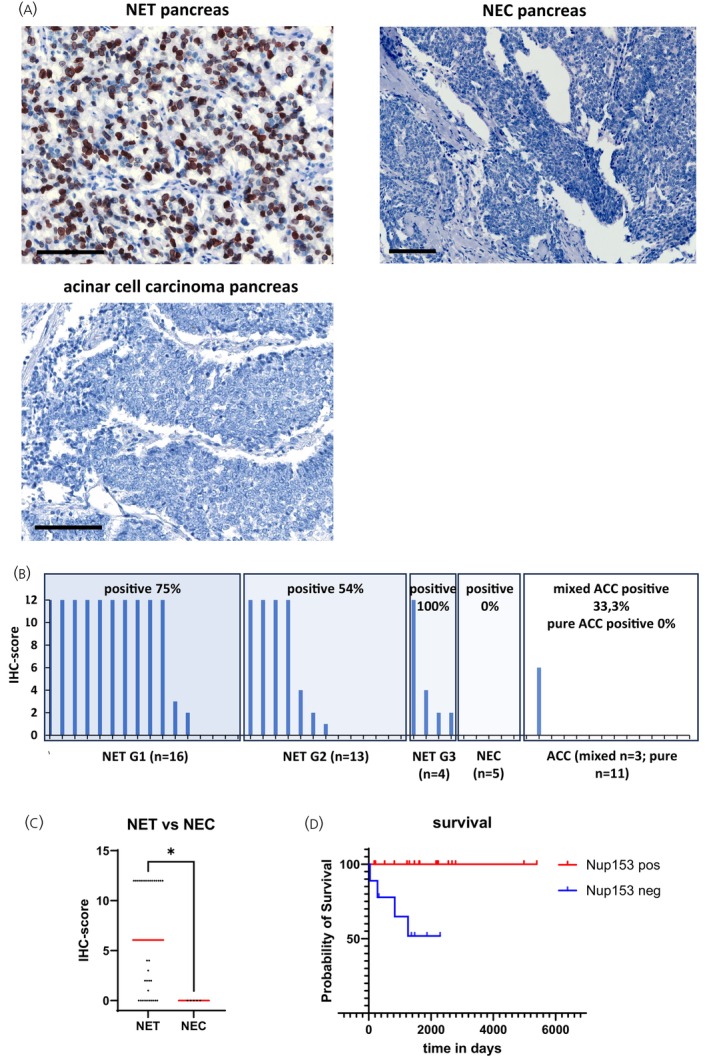
Nucleoporin‐153 (NUP153) immunoreactivity is significantly stronger in pancreatic neuroendocrine tumors (NETs) than in neuroendocrine carcinomas (NECs) and acinar cell carcinomas of the pancreas and is negatively correlated to survival. (A) Immunohistochemistry (IHC) of NUP153 in representative tissue specimen of NET (upper left panel), NEC (upper right panel) and acinar cell carcinomas (lower panel) of the pancreas. Scale bar, 100 μm. (B) Immunohistochemical scores of NUP153 staining in 33 NETs, five NECs, three mixed, and 11 pure acinar cell carcinomas of the pancreas. (C) IHC‐score of NUP153 in NET versus NEC (Mann–Whitney test, *p* < .05). (D) IHC‐score of the pNETs with regard to survival (*p* < .01). Significance level *p < .05, **p < .01, ***p < .001, ****p < .0001.

In pancreatic (p) NETs NUP153 positivity was observed in 69.7% of NETs G1‐G3 (23/33) compared to 0% of NECs (0/5) and 0% (0/11) of pure acinus cell carcinomas (Figures 3A,B and S1a). Accordingly, pNETs G1‐G3 showed significantly higher NUP153 immunoreactivity compared to pNECs (*p* = .012) as shown in Figure 3C. The survival of pancreatic neuroendocrine tumor (pNET) patients was positively correlated with NUP153 immunoreactivity (Figure 3D) with poorest outcome in patients with negative NUP153 staining compared to a better outcome with NUP153 positivity (*p* < .01). We found no differences in NUP153 immunopositivity with respect to tumor stage, patient age, sex, or tumor localization (Figure S1b–e).

**FIGURE 4:**
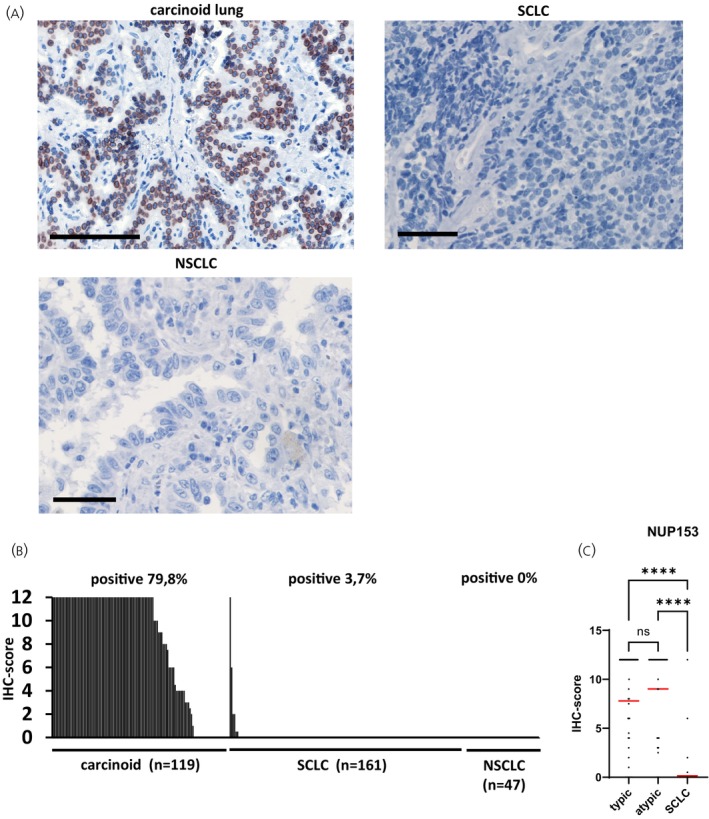
Nucleoporin‐153 (NUP153) immunopositivity is largely preserved in typical and atypical carcinoids of the lung compared to small cell lung carcinomas (SCLC), and non‐small cell lung carcinomas (NSCLC). (A) Immunohistochemistry (IHC) of NUP153 in representative tissue specimen of carcinoids (upper left panel, scale bar 100 μm), SCLCs (upper right panel, scale bar 50 μm), and NSCLCs (lower panel, scale bar 60 μm) of the lung. (B) Immunohistochemical scores of NUP153 staining in 119 carcinoids, 161 SCLCs, and 47 NSCLCs of the lung. (C) Immunohistochemistry (IHC)‐score of NUP153 compared in typic and atypic carcinoids of the lung and SCLCs (Dunn's multiple comparisons test, *p* < .0001). Significance level *p < .05, **p < .01, ***p < .001, ****p < .0001.

We then tested if these findings can be transferred to lung NEN, including typical and atypical carcinoids, small cell lung carcinomas (SCLC) and non‐small cell lung carcinomas (NSCLC). Indeed, as illustrated in Figure 4A,B, the vast majority (~80% [95/119]) of well‐differentiated rather slow proliferating (≤10 mitoses per 2 mm^2^) pulmonary NET (=typical and atypical carcinoids) were positive for NUP153, while the highly proliferating and extremely aggressive SCLCs were almost all completely negative (Figure 4A–C). In addition, the non‐NECs were also completely negative (0%, [0/47]), as described for the aforementioned organs. We found no differences in NUP153 immunopositivity with respect to patient sex, age, or lymph node metastases (Figure S2a–c).

We conclude from these data that NUP153 immunoreactivity is inversely correlated with tumor aggressiveness in NEN.

To exclude that long‐term storage of the FFPE material may have introduced age‐related staining artifacts, we analyzed the staining characteristics of all lung carcinoid cases from 2005 and 2007 (10 patients per year) and pNET cases from the 1990s (*n* = 9). As shown in Figures S5a–c all years included cases with minimum (0), intermediate, and maximum (12) IHC scores, providing no evidence that specimen age systematically influenced the staining results. Additionally, full‐section slides from three pNET G3 cases were stained and included in the analysis. Regardless of storage time (ranging from 2016 to 2022), all three tumors demonstrated positive stainings to a various degree (including subsets of negative tumor cells). These findings suggest that the observed staining results are not primarily attributable to storage‐related effects, as shown in Figure S1a. Nevertheless, a potential impact of long‐term FFPE storage on antigenicity cannot be completely excluded.

### 3.4 NUP153 immunoreactivity does not reflect total NUP153 protein abundance as measured by targeted mass spectrometry

**FIGURE 5:**
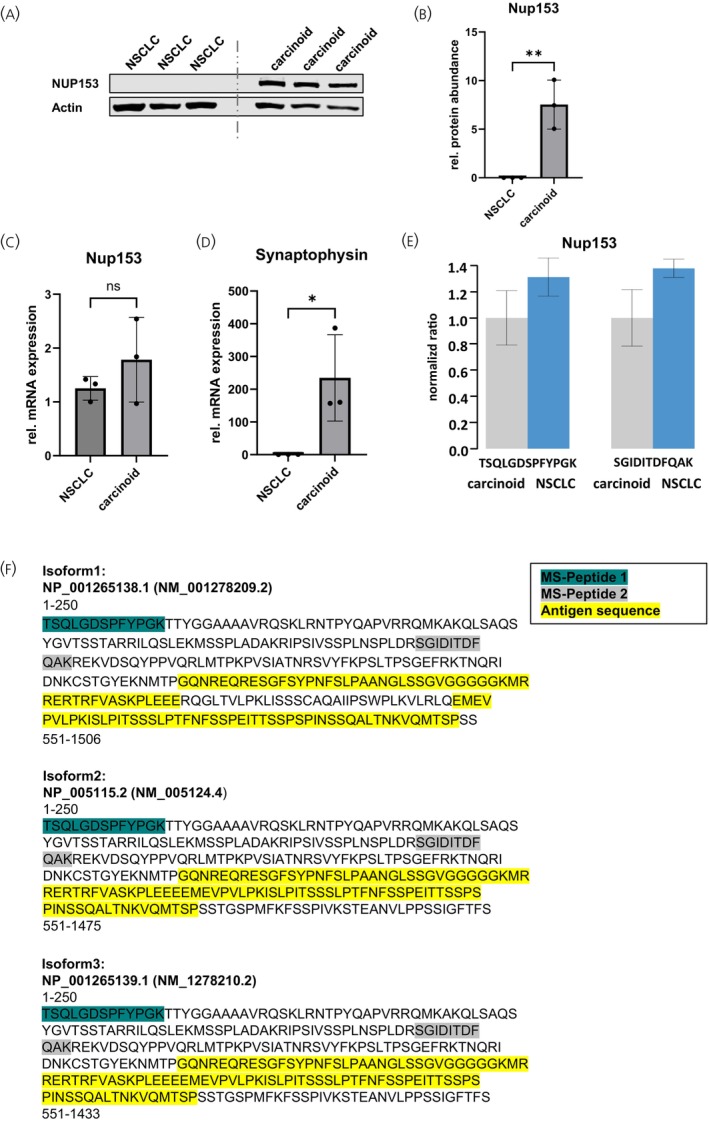
Nucleoporin‐153 (NUP153) signal by immunoblotting is present in lung carcinoids but absent in non‐small cell lung carcinomas (NSCLC), despite similar overall NUP153 mRNA and NUP153 protein expression levels. (A) Representative immunoblot analysis demonstrates strong NUP153 protein bands in lung carcinoids, minimal/no signal in NSCLC. Actin served as loading control. (B) Quantification of immunoblot signals reveals significantly higher NUP153 protein abundance in neuroendocrine tumors (NETs) compared to NSCLC. (C) qPCR analysis shows no significant difference in total NUP153 mRNA expression between NSCLC and NETs. (D) Synaptophysin mRNA expression, included as neuroendocrine marker and positive control is detected at high levels in lung carcinoids while being almost undetectable in NSCLC. (E) As an antibody‐independent approach targeted proteomics of the same samples (as in A–D) demonstrates no significant difference in NUP153 total protein expression between lung carcinoids and NSCLC. (F) Schematic alignment of NUP153 isoforms 1–3 shows peptide sequences detected by targeted mass spectrometry (gray and dark green) and the antigen sequence (yellow, provided by the company “Atlas Antibodies”). The uninterrupted antigen sequence is present in isoforms 2 and 3 but disrupted in isoform 1. Significance level *p < .05, **p < .01, ***p < .001, ****p < .0001.

Considering the well‐reported essential role of NUP153 in NPC function, nucleo‐cytoplasmic transport, mitotic spindle assembly, and regulation of gene expression (in addition to the embryonic lethality observed in NUP153 knockout models^26^) it appeared barely conceivable that all these critical cellular functions could be compensated in complete absence of NUP153 expression. To rule out the possibility that the observed selective NUP153 staining pattern is the result of an artifact by the formalin fixation and paraffin embedding procedure (FFPE) we chose immunoblotting of lysates derived from fresh‐frozen tumor tissue as an alternative approach. As shown in Figure 5A,B immunoblotting using lung carcinoid and NSCLC patient tissue samples revealed a strong band at the expected height in the carcinoid samples and no detectable NUP153 signal in the NSCLC samples and thereby complete consistency with the IHC result (uncropped western blot membrane in Figure S3). We then tested if this difference observed by immunoblotting was due to differences in messenger ribonucleic acid (mRNA) expression. However, NUP153 expression by quantitative real‐time polymerase chain reaction (qRT‐PCR) was not significantly different in carcinoids vs. NSCLC (Figure 5C), while expression of Synaptophysin was, as expected, strikingly positive in the carcinoids, but negative in the NSCLC (Figure 5D). We then applied a targeted proteomic approach selected reaction monitoring (SRM) as an antibody‐independent detection and quantification method using two different NUP153 peptides and again observed no significant difference in the abundance of NUP153 protein using the same carcinoid and NSCLC samples (Figure 5E).

We conclude from these data that there is no significant difference in total NUP153 mRNA or protein expression between well‐differentiated pulmonary NET and NSCLC. This in turn indicates that NUP153 immunoreactivity using the HPA27896 NUP153 antibody does not reflect overall NUP153 protein abundance as evidenced by an antibody‐independent targeted MS approach.

### 3.5 Differences in NUP153 immunoreactivity are independent of isoform expression and likely reflect post‐translational modifications

**FIGURE 6:**
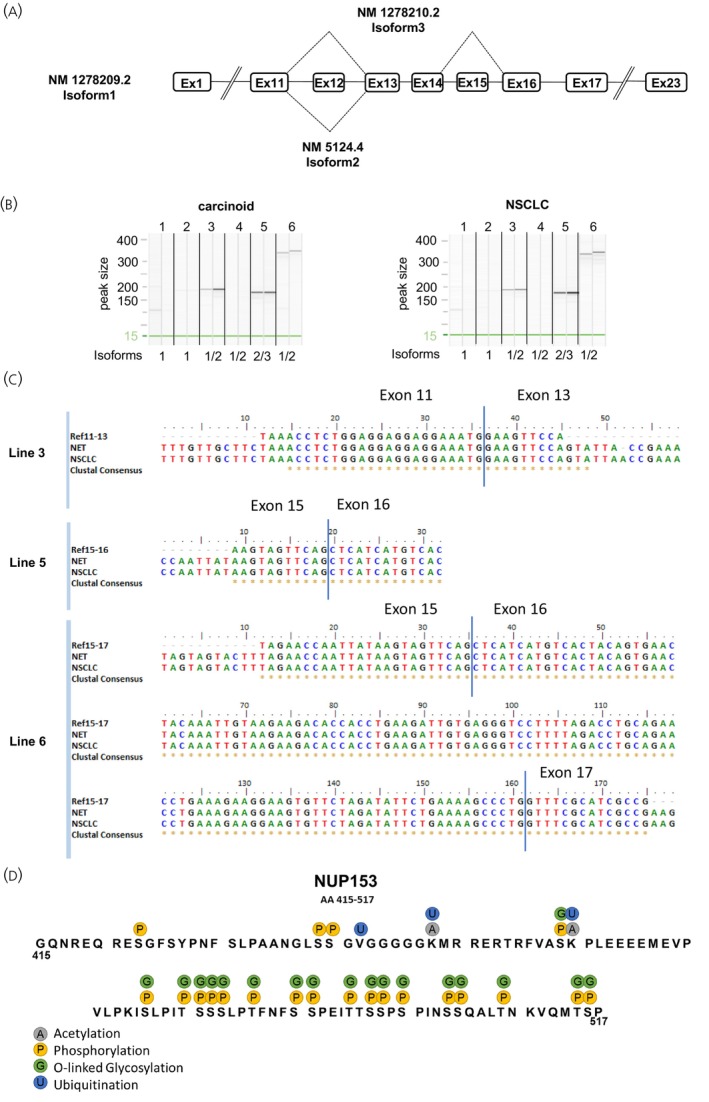
Carcinoids and non‐small cell lung carcinomas (NSCLC) both express nucleoporin‐153 (NUP153) isoform 2—posttranslational modifications (PTM) mapping from databases indicate a high PTM density in the antigen sequence. (A) Graphical representation of the exon distribution of the different isoforms of NUP153. (B) Gel electrophoretic separation of the intron‐spanning PCR products relevant for differentiating the isoforms. Lanes 1–6 show PCR products in duplicates. Size ladder is depicted in base pairs (bp). 1: Exon 11–12,198 bp, 2: Exon 12–13,185 bp, 3: Exon 15–16,189 bp, 4: Exon 16–17,170 bp, 5: Exon 11–13,175 bp, 6: Exon 15–17,191 bp (340 bp including exon 16). (C) Sequencing results of the described PCR products. (D) Amino acid sequence of nuclear pore complex protein NUP153_HUMAN from amino acids 415–517 including the antigen sequence with experimentally validated PTMs reported in the dbPTM database (https://awi.cuhk.edu.cn/dbPTM/index.php).

We then hypothesized that the NUP153 antibody detects a specific NUP153 isoform being present in well‐differentiated NETs and not expressed in NECs or non‐NECs. The three reported NUP153 isoforms are displayed in Figure 6A. In fact, the full length and uninterrupted antigen sequence (see also Figure 5F, highlighted in yellow) of the NUP153 antibody (HPA27896) is only present in the NUP153 protein of isoform 2 (NM 5124.4) and isoform 3 (NM 1278210.2), but not in isoform 1 (NM 1278209.2). In contrast, the two NUP153 MS peptides used for targeted proteomics detect all three NUP153 protein isoforms (Figure 5F, highlighted in gray and green, respectively). To further discriminate between the respective isoforms, we designed primer pairs resulting in intron‐spanning polymerase chain reaction (PCR) products covering different exon junctions. There was no convincing PCR product at the expected size containing the Exon 11–12 (1) and Exon 12–13 (2) junction as well as the Exon 16–17 (4) junction, neither in the carcinoid nor in the NSCLC samples indicating that both tumor types do not express isoform 1 (NM 1278209.2) (Figures 6B and S4). However, the detectable PCR product at the expected size containing Exon 15–16 junction (3) Exon 11–13 junction (5) and Exon 15–17 (including Exon 16) suggest that both NET and NSCLC express isoform 2 (NM 5124.4) (Figure 6B). The corresponding sequencing results of the PCR products are in line with the respective reference sequences as indicated (Figure 6C).

By exclusion we finally hypothesized that post‐translational modifications (PTMs) might cause the observed differences in NUP153 immunoreactivity. To further explore this possibility, we queried the dbPTM database (https://awi.cuhk.edu.cn/dbPTM/index.php) using the antigen sequence NUP153 (HPA27896). This analysis revealed a high density of experimentally validated PTMs within the antigen sequence, including 21 phosphorylation sites, 18 O‐glycosylation sites, three ubiquitination sites, and two acetylation sites, indicating that the antibody apparently binds to a heavily modified segment of the protein (Figure 6D). In the absence of differences in total NUP153 protein abundance or isoform composition, these PTMs represent the most likely explanation for the differential IHC reactivity (HPA27896) observed between well‐differentiated NETs and either NECs or non‐NECs (see also Section 4).

## 4 DISCUSSION

In the present study, we identified a largely selective immunohistochemical positivity for NUP153 (HPA27896) in neuroendocrine tissues/cells being widely retained in corresponding well‐differentiated NETs. NUP153 immunoreactivity represented a marker for favorable prognosis. Importantly, our data suggest that this selective immunohistochemical detection is neither explained by differences in NUP153 mRNA nor total NUP153 protein levels or differential isoform usage, but most likely reflects PTMs affecting the antibody epitope.

Interestingly, the initial comparative NUP98/NUP153 screening approach of different tissues and cell types not only indicated neuroendocrine cells but also neurons, Sertoli cells of the testis, and distal nephron epithelial cells to be positive for NUP153. This leads to the question if these cells, despite being very diverse, may share key biological properties. Originating from different germ layers (neuroectoderm for neurons and many neuroendocrine cells, mesoderm for Sertoli and renal epithelial cells), they converge functionally as highly differentiated and specialized cells with low‐proliferative potential. Many of them are long‐lived, hormonally regulated/hormone producing, or secretory in nature and rely on tightly maintained gene expression programs. These characteristics may necessitate a specialized form of nuclear organization favoring a NPC configuration/modification optimized for chromatin stability and tightly regulated nuclear transport. The consistent NUP153 immunoreactivity in these cell types may therefore reflect a shared NUP153‐related NPC phenotype which requires (or is required for) a high degree of differentiation. A phenotype that in the case of NENs is largely maintained in well‐differentiated NETs (G1–G3) and nearly completely lost in NECs.

These results are in line with the findings of Simon et al.^27^ By using a methylation analysis of 57 PanNEN samples, they demonstrated that NECs exhibit a distinct methylation profile suggestive of an exocrine cell of origin, thereby supporting a separate cellular lineage compared to other endocrine‐derived PanNENs. This provides a possible explanation for the observed lack of Nup153 immunoreactivity in the exocrine pancreas, including acinar cell carcinomas, as well as in poorly differentiated neuroendocrine carcinomas (pNECs). In contrast, Nup153 positivity was consistently present in Islets of Langerhans and was detected in the majority of well‐differentiated pancreatic neuroendocrine tumors (pNET G1–G3). We consider the distinct cellular lineage and differentiation state to be the more likely explanation for the observed Nup153 immunoreactivity pattern than differences in Ki67 proliferation activity alone.

NUP153, as a major component of the nuclear basket, is known to participate in chromatin tethering, transcriptional regulation, and nuclear envelope reassembly, and these functions have been demonstrated to involve PTMs. During mitosis, peripheral NUPs such as NUP153, NUP214, and NUP358 are subjected to extensive hyperphosphorylation, facilitating NPC disassembly and reassembly in synchrony with the cell cycle as reviewed by Rabut et al.^28^ Beyond mitosis, PTMs have also been documented to shape NPC structure and function in non‐dividing cells. In yeast, the NUP153 orthologue NUP60 undergoes SUMOylation and monoubiquitination, which modulate its dynamic association with the NPC and impact DNA damage responses and telomere anchoring, as described by Nino et al.^29^ Moreover, studies by Ori et al.^30^ and others have demonstrated that NPC composition varies between cell types, with stoichiometric differences reflecting distinct functional demands and possibly driven by PTMs.

Several studies have shown that PTMs such as phosphorylation and acetylation regulate NUP153's binding interactions and localization. For example, Kosako et al. could show that extracellular signal‐regulated kinase (ERK) phosphorylates NUP153 affecting the interaction with importin‐β^31^ and McCloskey et al. demonstrated that ERK‐mediated phosphorylation modulates NUP153's interaction with the NUP107 complex,^32^ affecting pore assembly. Recent phosphoproteomics studies also highlight the differences in the PTM pattern in NUP153. The phosphoproteomic analysis of non‐functional pancreatic neuroendocrine tumors (NF‐PanNETs) by Ji et al.^33^ which used 108 fresh‐frozen NF‐PanNET samples and paired normal adjacent tissues, revealed not only globally low phosphorylation levels but also similar observations for the nuclear pore component proteins, such as NUP153. In contrast, phosphoproteomic studies of carcinoma cell lines exhibit the presence of a large number of phosphosites on NUP153,^34^, ^35^ including the antigen sequence region. Therefore, it seems conceivable that also NUP153‐related functions in the above‐mentioned cell types and tumors require specific NUP153 PTMs which in turn determine epitope accessibility of the used NUP153 antibody.

Beyond NPC components and consistent with the idea of PTM‐mediated epitope masking, Lee et al. demonstrated that removal of N‐linked glycosylation significantly improves anti‐PD‐L1 antibody binding affinity and IHC signal intensity^36^ and Mei et al. similarly noted that deglycosylation enhances programmed death‐ligand 1 (PD‐L1) detection.^37^ Likewise, Maya and Oren^38^ could show that phosphorylation can block antibody recognition of P53 and that phosphatase treatment unmasks phosphorylation‐sensitive epitopes. In the context of NUP153, the dense clustering of PTMs within the antigen sequence may suggest a similar scenario in which PTMs may sterically block the antibody binding sites, depending on cell state and lineage. Commercial antibodies are typically raised against unmodified peptide sequences, meaning that modification of the corresponding site in the native protein, such as phosphorylation, can prevent antibody binding. In contrast, generation of a (for instance) phospho‐specific antibody requires deliberate immunization with a phosphorylated peptide and rigorous validation for modification specificity. This in turn makes it quite unlikely that the used NUP153 antibody is inadvertently phospho‐specific (or specific for other PTMs). Our data therefore support a model in which increased (multiple) PTMs in aggressive tumors impede epitope recognition instead of reflecting the absence of the protein.

In a diagnostic setting, regardless of the exact cause of the selective NUP153 immunoreactivity, NUP153 (HPA27896) positivity could be considered a complementary diagnostic marker in addition to established neuroendocrine markers such as synaptophysin, chromogranin A, or insulinoma‐associated protein 1 (INSM1). However, validation in larger, independent cohorts will be essential to (a) further define the clinical utility of NUP153 IHC in the differential diagnosis of neuroendocrine neoplasms and (b) more broadly, explore PTM‐related NPC changes in these tumors.

## AUTHOR CONTRIBUTIONS

The main contributions by authors are as follows: study concept and design (KS and SM), acquisition, analysis and interpretation of the data (KS, SM, AA, VH, IB, SS, EB, KK, and KB), resources (FB, EH, TM, MM, JL‐K, and AW), writing and editing of the original draft (SM, KS, SS, VH, AA, BG, and IB).

## FUNDING INFORMATION

Kerstin Singer received a fellowship by the Rahel‐Goitein‐Straus Program of the Medical Faculty Heidelberg, support from the Junior Academy of the German Society for Pathology, as well as support from the TÜFF Habilitation program of the Medical Faculty Tübingen. Stephan Singer was supported by Deutsche Forschungsgemeinschaft (DFG) grant Si1487/3‐1, TRR/SF209 (B04), by the Hella‐Buehler‐Foundation and by a HRCMM (Heidelberg Research Center for Molecular Medicine) Career Development Fellowship.

## CONFLICT OF INTEREST STATEMENT

There are no competing interests to disclose.

## ETHICS STATEMENT

The use of the patient tissue was approved by the local ethics committee of the University Hospitals in Heidelberg and Tübingen, respectively, with the following approval numbers S‐206/2005, 508/2016BO1, and 032/2021B02.

## Supporting information


**Data S1.** Supporting Information.

**Data S2.** Supporting Information.

## Data Availability

The datasets generated and/or analyzed during the current study are available upon reasonable request.
