# Supplementary material for: NUP153 immunoreactivity in neuroendocrine neoplasms supports the diagnosis of NET versus NEC and is modulated by post‐translational modifications
Source: J Neuroendocrinol. 2026 Aug 12;38(8):e70240. doi: 10.1111/jne.70240 (PMC13468175; doi:10.1111/jne.70240)
Supplement: Supplementary file 1 [file JNE-38-e70240-s001.docx]

Supplement Information

**MATERIAL & METHODS**

**Patient samples and tumor specimens**

For the immunohistochemical (IHC) screening of normal tissues, pan-normal tissue microarrays (TMAs) comprising more than 25 healthy tissue types (table S1 and Table S2) were used. The exact number depends on the section level.

| **human tissue/organ** | **total number** | **patient characteristics** | | |
| --- | --- | --- | --- | --- |
|  |  |  |  |  |
|  |  | **age (mean)** | **m** | **f** |
| **Adrenal gland** | **2** | **40** | **2** | **0** |
| **Brain** | **1** | **60** | **0** | **1** |
| **Colon** | **16** | **57.8** | **5** | **11** |
| **Eosophagus** | **13** | **65.5** | **8** | **5** |
| **Gall bladder** | **8** | **57** | **5** | **3** |
| **Heart** | **13** | **68** | **8** | **5** |
| **Kidney** | **16** | **52.4** | **10** | **6** |
| **Liver** | **12** | **59.1** | **10** | **2** |
| **Lung** | **10** | **50.1** | **5** | **5** |
| **Muscle** | **7** | **53.7** | **4** | **3** |
| **Ovary** | **6** | **66.3** | **0** | **6** |
| **Pancreas** | **7** | **48.1** | **4** | **3** |
| **Prostate** | **13** | **68.5** | **13** | **0** |
| **Salivary gland** | **8** | **61** | **6** | **2** |
| **Skin** | **3** | **63.7** | **0** | **3** |
| **Small bowel** | **13** | **57.1** | **5** | **8** |
| **Spleen** | **14** | **56.7** | **3** | **11** |
| **Stomach** | **13** | **60.9** | **6** | **7** |
| **Testis** | **12** | **66.2** | **11** | **1** |
| **Thymus** | **7** | **32.2** | **4** | **3** |
| **Thyroid gland** | **11** | **45** | **4** | **7** |
| **Lymphatic tissue** | **9** | **31.4** | **7** | **2** |
| **Urinary bladder** | **4** | **64** | **2** | **2** |
| **Uterus** | **5** | **70** | **0** | **5** |
| **White adipose tissue** | **10** | **56.1** | **8** | **2** |

Table S1: Composition of Pan-Tissue-Array for Nup98 staining

Table S2: Composition of Pan-Tissue-Array for NUP153 staining

| **human tissue/organ** | **total number** | **Patient characteristics** | | |
| --- | --- | --- | --- | --- |
|  |  |  |  |  |
|  |  | **age (mean)** | **m** | **f** |
| **Adrenal gland** | **3** | **40.3** | **3** | **0** |
| **Brain** | **1** | **60** | **0** | **1** |
| **Colon** | **14** | **55.1** | **5** | **9** |
| **Eosophagus** | **13** | **65.5** | **8** | **5** |
| **Gall bladder** | **7** | **59** | **4** | **3** |
| **Heart** | **12** | **68.5** | **7** | **5** |
| **Kidney** | **11** | **50.3** | **6** | **5** |
| **Liver** | **11** | **58.2** | **9** | **2** |
| **Lung** | **10** | **50.1** | **5** | **5** |
| **Muscle** | **5** | **55** | **3** | **2** |
| **Ovary** | **5** | **70** | **0** | **5** |
| **Pancreas** | **2** | **37.5** | **1** | **1** |
| **Prostate** | **13** | **68.5** | **13** | **0** |
| **Salivary gland** | **6** | **61.5** | **5** | **1** |
| **Skin** | **2** | **49** | **0** | **2** |
| **Small bowel** | **12** | **57.2** | **4** | **8** |
| **Spleen** | **12** | **53.4** | **1** | **11** |
| **Stomach** | **12** | **60.1** | **6** | **6** |
| **Testis** | **12** | **66.2** | **11** | **1** |
| **Thymus** | **5** | **34.8** | **4** | **1** |
| **Thyroid gland** | **8** | **46.6** | **2** | **6** |
| **Lymphatic tissue** | **8** | **33.4** | **2** | **6** |
| **Urinary bladder** | **4** | **64** | **1** | **3** |
| **Uterus** | **2** | **68.5** | **0** | **2** |
| **White adipose tissue** | **7** | **51.9** | **6** | **1** |

Archival specimens, collected between 1992 and 2006 at the Pathology Institute of the University Hospital Heidelberg for diagnostic purposes, were formalin-fixed, paraffin-embedded (FFPE), and stained with hematoxylin and eosin (H&E). Slides were independently re-evaluated by at least two pathologists to identify representative areas. Based on these assessments, cylindrical cores (1 mm in diameter) were extracted from donor blocks using a TMA instrument (Beecher MTA-1, Beecher Instruments, Inc., Sun Prairie, USA) and arrayed in a reproducible manner into recipient blocks. This procedure was applied to all TMAs described below.

The TMAs of small bowel and appendix tumors included samples from patients who underwent surgery at the University Hospital Heidelberg or affiliated hospitals between 1995 and 2011 (for detailed TMA information see Table 3). Diagnoses were established according to the most recent World Health Organization criteria. Representative areas containing >50% viable tumor, were selected. In most cases, two cores were included, while in small tumors only one was feasible due to limited tissue.

The pancreatic TMAs contained predominantly two or more representative cores per tumor. Again, in small lesions only one core was possible (for detailed TMA information see Table S3). Construction was performed as previously described (1, 2).

The lung TMAs included one or two representative cores per tumor, depending on tumor size. The lung neuroendocrine neoplasms (NEN) TMAs were generated as reported previously (3), while the non-small cell lung cancer (NSCLC) TMA was constructed as described above (for detailed TMA information see table S3).

Table S3: Patient characteristics of aforementioned TMAs and included full section slides.

**Patient samples used for Immunoblotting, Mass Spectrometry and qPCR**

The fresh frozen tumor tissue from lung carcinoids and NSCLCs were obtained from patients who underwent resection for lung cancer at the Department of Thoracic Surgery, Thoraxklinik Heidelberg-Rohrbach (Germany). Informed consent was obtained from all patients and/or their legal guardian(s). All methods were carried out in accordance with relevant guidelines and regulations. Each tumor sample was snap-frozen in liquid nitrogen within 30 min after resection and stored at −80°C. Samples were stained with H&E and carefully reviewed by a pathologist to determine the proportion of viable tumor cells, stromal cells, normal lung cell content, infiltrating lymphocytes, and necrotic areas. Only sections with ≥50% viable tumor cells were considered for further analyses.

**Immunohistochemistry and tissue microarray analysis**

Immunohistochemical staining was evaluated using a composite score calculated by multiplying staining intensity and the proportion of positive tumor cells for each TMA tissue sample. Staining intensity was scored as 0 (negative), 1 (weak), 2 (moderate), or 3 (strong), whereas the proportion of positive tumor cells was scored as 0 (no expression), 1 (<1%), 2 (<10%), 3 (10–50%), or 4 (>50%). In the case that a tumor is represented by several cores, the reported value represents the median of the results. Immunohistochemical staining was performed with a commercially available rabbit polyclonal anti-NUP153 antibody (HPA027896, Atlas Antibodies AB, Stockholm, Sweden) in a 1:100 dilution (Dako REAL Antibody Diluent, Dako, Hamburg, Germany) after a well-established protocol. First step was the deparaffinization and rehydration of each TMA slide by passing through multiple washing steps: 2x 10 min Xylene, 2x 5 min 100% ethanol, 1x 5 min 96% ethanol, 1x 5 min 70% ethanol, ending with distilled water. Heat-induced epitope retrieval is achieved by a 10 min pretreatment in a pressure cooker using REAL Target Retrieval Solution on pH6 (Dako, Hamburg, Germany) followed by a thirty min cool down. For primary and secondary antibody incubation as well as staining procedures slides are transferred to an automated immunostaining instrument (Dako Autostainer, Dako, Hamburg, Germany) which detection system is based on a labelled streptavidin-biotin method (Dako REAL Detection System Peroxidase/AEC Rabbit/mouse, Dako, Hamburg, Germany) by passing through the following steps: 30 min incubation with the primary antibody, rinsing in PBS/Tween, 20 min treatment with biotinylated goat anti-rabbit secondary antibody, rinsing in PBS/Tween, H_2_O_2_ incubation for 5 min, rinsing in PBS/Tween, exposure to HRP for 20 min rinsing in PBS/Tween, 7 min incubation with 3-amino-9-ethylcarbazole/H2O2, repetition of the last two steps, rinsing in PBS/Tween and finally distilled water. Hematoxylin was used for a 5 min counterstaining step followed by a 5 min wash in tap water. Capping the slides with cover slips was performed by using Aquatex mounting media (Merck, Darmstadt, Germany).

NUP98 rabbit polyclonal antibody was kindly provided by the laboratory from Dr. G. Blobel (Rockefeller University, NYC, USA). Immunohistochemical staining was performed corresponding to NUP153.

**Gel electrophoresis and immunoblotting**

One hundred milligrams of the sample were crushed in cell lysis buffer (New England Biolabs), centrifuged at 18,000 × *g* (4°C, 15 min), and quantified using the Bradford assay. After boiling samples for 5 min at 90°C, proteins were separated by SDS/PAGE and transferred to nitrocellulose membranes (Whatman, Dassel, Germany). Membranes were blocked with 5% milk/TBST for 1h and incubated overnight with anti-NUP153 (HPA027896, Atlas Antibodies AB, Stockholm, Sweden) diluted in the same blocking solution at 1:500. The detection of ß-actin verified an equal loading of each protein sample by using a monoclonal mouse anti-actin antibody in a dilution 1:10000 (MP Biomedicals, Illkirch, France). After rinsing with TBST the membranes were incubated for 1h with the corresponding fluorescent-secondary antibodies. After rewashing with TBST detection was performed using Odysee Sa Infrared Imaging System (LI-COR Bioscience, Bad Homburg, Germany).

**Total RNA isolation, cDNA synthesis and quantitative real-time polymerase chain reaction (qPCR)**

Total RNA was isolated using the RNeasy Mini Kit (Qiagen). The quantity of RNA was measured with a NanoDrop ND-1000 Spectrophotometer and the quality of total RNA was assessed with Agilent 2100 Bioanalyzer (Agilent Technologies). Samples were considered sufficient for further analysis if the RNA integrity number was ≥8.0. Complementary DNA (cDNA) was generated from 1 µg of RNA by reverse transcription with the Sigma-Aldrich RT-PCR kit following the manufacturer’s recommendations. Quantitative real-time PCR was performed on a StepOnePlus system (Applied Biosystems, Darmstadt, Germany) employing the PrimaQUANT qPCR CYBR-Green-MasterMix-high-ROX (Steinbrenner, Heidelberg, Germany). Each reaction was carried out in technical duplicates. Gene expression values were normalized to RPL32 expression using the ΔΔCt calculation method. Primers were custom-designed and synthesized by ThermoFisher Scientific (Offenbach, Germany) with the sequences as listed in table S4.

| NUP153#F | CCCTCTCCACCCTTACGTCT |
| --- | --- |
| NUP153#R | GCCTCCAAAATAAAGGCGGG |
| Synaptophysin#F | TGGGGACTACTCCTCGTCAG |
| Synaptophysin#R | AGAGCCCCCATGGAGTAGAG |
| L32#F | TTCCGGTCCACAACGTCAAG |
| L32#R | TGTGAGCGATCTCGGCAC |

Table S4: Primer sequences for qPCR

**NUP153 mRNA isoform analysis**

500 ng RNA were transcribed into cDNA using Superscript II reverse transcriptase (Thermo Fisher Scientific, Waltham, MA, USA) and random hexamers (Thermo Fisher Scientific) in a final volume of 20 ml according to the manufacturer’s instructions. NUP153 isoforms were investigated by next generation sequencing with single amplicons using the Ion Amplicon Library Preparation Fusion Method (ThermoFisher Scientific) according to the manufacturers protocol. PCRs were carried out using 1 µM Primers (table S5) using the following settings (94°C 3 min; 40 cycles at 94°C 30 sec, 58°C 30 sec and 68°C 1 min). PCR products were visualized using the Qiaxcel Advanced platform (Qiagen, Hilden, Germany). Amplicons were purified and quantified applying Agencourt AMPure XP (Beckman Coulter, Brea, CA, USA) magnetic beads and the Qubit dsDNA HS Assay Kit (Thermo Fisher Scientific). Amplicons were diluted to 5 pM each and pooled. Clonal amplification and semiconductor sequencing was done according to the manufacturer’s manuals using the Ion 540 Kit – Chef on the Ion Chef Instrument and the Ion 540 Chip Kit on the Ion GeneStudio S5 Prime system (Thermo Fisher Scientific). FASTQ Files were generated with Torrent Suite.

Table S5: Primer sequences for PCR

| NUP153_Ex11-12_BC60_F | CCATCTCATCCCTGCGTGTCTCCGACTCAGTCTAGCTCTTCGATTGGAGGTGGCAAGATGAGAC |
| --- | --- |
| NUP153_Ex11-12_BC60_R | CCATCTCATCCCTGCGTGTCTCCGACTCAGTCTAGCTCTTCGATAGATGGGATGATCGCTTGAG |
| NUP153_Ex11-12_trP1_F | CCTCTCTATGGGCAGTCGGTGATTGGAGGTGGCAAGATGAGAC |
| NUP153_Ex11-12_trP1_R | CCTCTCTATGGGCAGTCGGTGATAGATGGGATGATCGCTTGAG |
| NUP153_Ex12-13_BC61_F | CCATCTCATCCCTGCGTGTCTCCGACTCAGTCACTCGGATCGATCTCAAGCGATCATCCCATCT |
| NUP153_Ex12-13_BC61_R | CCATCTCATCCCTGCGTGTCTCCGACTCAGTCACTCGGATCGATAATTAAAGGTAGGCAGTGAAGAAC |
| NUP153_Ex12-13_trP1_F | CCTCTCTATGGGCAGTCGGTGATTCAAGCGATCATCCCATCT |
| NUP153_Ex12-13_trP1_R | CCTCTCTATGGGCAGTCGGTGATAATTAAAGGTAGGCAGTGAAGAAC |
| NUP153_Ex15-16_BC62_F | CCATCTCATCCCTGCGTGTCTCCGACTCAGTTCCTGCTTCACGATTGTACTACCTCCATCATCTATTGG |
| NUP153_Ex15-16_BC62_R | CCATCTCATCCCTGCGTGTCTCCGACTCAGTTCCTGCTTCACGATGTGACATGATGAGCTGAACTACT |
| NUP153_Ex15-16_trP1_F | CCTCTCTATGGGCAGTCGGTGATTGTACTACCTCCATCATCTATTGG |
| NUP153_Ex15-16_trP1_R | CCTCTCTATGGGCAGTCGGTGATGTGACATGATGAGCTGAACTACT |
| NUP153_Ex16-17_BC63_F | CCATCTCATCCCTGCGTGTCTCCGACTCAGCCTTAGAGTTCGATAAGATTGTGAGGGTCCTTTTAGA |
| NUP153_Ex16-17_BC63_R | CCATCTCATCCCTGCGTGTCTCCGACTCAGCCTTAGAGTTCGATGAATCTATCTTCGGCGATGCG |
| NUP153_Ex16-17_trP1_F | CCTCTCTATGGGCAGTCGGTGATAAGATTGTGAGGGTCCTTTTAGA |
| NUP153_Ex16-17_trP1_R | CCTCTCTATGGGCAGTCGGTGATGAATCTATCTTCGGCGATGCG |
| NUP153_Ex11-13_BC64_F | CCATCTCATCCCTGCGTGTCTCCGACTCAGCTGAGTTCCGACGATTGGAGGTGGCAAGATGAGAC |
| NUP153_Ex11-13_BC64_R | CCATCTCATCCCTGCGTGTCTCCGACTCAGCTGAGTTCCGACGATTGATCGGTAGAGAGATTTTCGGT |
| NUP153_Ex11-13_trP1_F | CCTCTCTATGGGCAGTCGGTGATTGGAGGTGGCAAGATGAGAC |
| NUP153_Ex11-13_trP1_R | CCTCTCTATGGGCAGTCGGTGATTGATCGGTAGAGAGATTTTCGGT |
| NUP153_Ex15-17_BC65_F | CCATCTCATCCCTGCGTGTCTCCGACTCAGTCCTGGCACATCGATGGCAAATGTACTACCTCCATCA |
| NUP153_Ex15-17_BC65_R | CCATCTCATCCCTGCGTGTCTCCGACTCAGTCCTGGCACATCGATATCTATCTTCGGCGATGCGA |
| NUP153_Ex15-17_trP1_F | CCTCTCTATGGGCAGTCGGTGATGGCAAATGTACTACCTCCATCA |
| NUP153_Ex15-17_trP1_R | CCTCTCTATGGGCAGTCGGTGATATCTATCTTCGGCGATGCGA |

**Analysis by targeted proteomics**

NUP153 was quantified in the samples utilizing targeted proteomics in conjunction with the spike-in of isotopically labeled peptides (JPT Peptide Technologies GmbH (Berlin, Germany) as described previously (4). Briefly, the assay was implemented on a TSQ Vantage (Thermo Fisher Scientific) coupled to Easy nLC II, using in-house packed PicoFrit emitters (75 μm ID, 10 μm tip, New Objective Inc., Woburn, MA, USA) with 11 cm of Magic C18 AQ resin (3 μm particle size, 200 Å pore size, Michrom Bioresources Inc., Auburn, CA, USA). The following gradient program was used to separate the 0.7 µg of peptides: 5–35% B in 30 min, 35–100% B in 2 min, and 100% B in 8 min, where A = 3% (v/v) acetonitrile in 0.1% (v/v) formic acid in water and B = 3% (v/v) water in 0.1% (v/v) formic acid in acetonitrile. The flow rate of 300 nL/min and spray voltage of 1.9 kV were used. LC-MRM assays were developed from previously available high-resolution LC-MS/MS data. MRM settings were as follows: 0.7 Da resolution, 2.5 s cycle time; collision energies vendor-calibrated. Data processed with mProphet; top 5 transitions per peptide selected. Scheduled MRM used with 3 min windows. Two proteotypic peptides, TSQLGDSPFYPGK and SGIDITDFQAK, were used for the quantification. Using the mProphet (Reiter et al., 2011) package, L/H ratios were computed from the apex sum of the chosen transitions (n=5).

**Statistical Analysis and Software**

Immunoreactivity differences in human tissue samples were assessed by nonparametric Mann-Whitney U-test, or Dunns’s multiple comparisons test using GraphPad Prism 10.1.1 (GraphPad Software, Inc, La Jolla, CA, USA). Scatter-plot with mean displayed in red.

Bar chart of relative mRNA expression and protein abundance are shown with standard deviations. Expression differences were assessed by unpaired t-test using GraphPad Prism 10.1.1 (GraphPad Software, Inc, La Jolla, CA, USA).

Overall survival analysis in patients was performed by Kaplan-Meier Curves and log-rank *p* values using GraphPad Prism 10.1.1 (GraphPad Software, Inc, La Jolla, CA, USA).

The statistical significance was defined as *p* < 0.05.

Photos of the stained TMAs were taken with IrfanView at different magnifications as specified.

**References**

1.	Gaida MM, Welsch T, Herpel E, Tschaharganeh DF, Fischer L, Schirmacher P, Hansch GM, Bergmann F. MHC class II expression in pancreatic tumors: a link to intratumoral inflammation. *Virchows Arch*. 2012; **460**(1): 47–60.

2.	Bergmann F, Aulmann S, Sipos B, Kloor M, von Heydebreck A, Schweipert J, Harjung A, Mayer P, Hartwig W, Moldenhauer G, Capper D, Dyckhoff G, Freier K, Herpel E, Schleider A, Schirmacher P, Mechtersheimer G, Klöppel G, Bläker H. Acinar cell carcinomas of the pancreas: a molecular analysis in a series of 57 cases. *Virchows Arch*. 2014; **465**(6): 661–72.

3.	Zahel T, Krysa S, Herpel E, Stenzinger A, Goeppert B, Schirmacher P, Hoffmann H, Schnabel PA, Warth A. Phenotyping of pulmonary carcinoids and a Ki-67-based grading approach. *Virchows Arch*. 2012; **460**(3): 299–308.

4.	Ori A, Banterle N, Iskar M, Andrés-Pons A, Escher C, Bui HK, Sparks L, Solis-Mezarino V, Rinner O, Bork P, Lemke EA, Beck M. Cell type-specific nuclear pores: a case in point for context-dependent stoichiometry of molecular machines. *Mol Syst Biol*. 2013; **9**.
